# TOR-inhibitors as gero-suppressors

**DOI:** 10.18632/aging.100869

**Published:** 2015-12-30

**Authors:** Mikhail Nikiforov

**Affiliations:** Department of Cell Stress Biology, Roswell Park Cancer Institute, Buffalo, NY 14263, USA

**Keywords:** mTOR, Rapamycin, mTOR inhibitors, gero-suppression

Rapamycin an inhibitor of TORC1 is the only proven potent inhibitor of aging, which extends lifespan in yeast, Drosophila (fly), warms, mice and potentially other mammals [1-5]. TOR is an enzyme discovered by Dr. Michael Hall the prominent American-Swiss scientist who received the most prestigious Breakthrough Prize in Life Sciences in 2014 for this discovery. The scope of his seminal finding exceeds this prize and more prizes will certainly follow. In mammals, mTOR includes two complexes: TORcomplex1 (TORC1) and TORcomplex2 (TORC2). The ability of Rapamycin to not only prolongs lifespan, but also delay diseases transforms this agent found on the Easter Island to one of the most famous molecules in the world. There are several analogs (e.g. everolimus (sirolimus), that target the same molecule (mTORC1) with variable potency and display some difference in biochemical properties. All these drugs termed rapalogs as well as Rapamycin will definitely become one of the most important scientific revolutions in the 21 century [6].

Needles to say that calorie restriction also inhibits TORC1, thus providing a possible explanation as to why calorie restriction extends lifespan in animals [7, 8]. On the other hand, calorie restriction inhibits TORC1 much less efficiently than rapamycin [8].

In addition unlike Rapamycin, calorie restriction or fasting may be difficult to implement in general population . Most importantly, Rapamycin has minimal side effects which is not always true for fasting due to loss of important nutrients that affect multiple pathways [7, 8]. Although rapalogs, including Rapamycin, show great promise, it will be tempting to search for anything that could increase the positive effects of rapalogs [9]. At first glance, it is impossible. For example, pan-TOR inhibitors, which inhibit all TOR-kinase complexes, including TORC1 and TORC2, will have all beneficial effects of TORC1 inhibition, but on the other hand will inhibit TORC2 as well, thus causing potential side-effects. Although for many years rapalogs have been considered the best in its class, recent years brought some pleasant surprises [9]. Thus, it was found that mTORins, dual mTOR kinase inhibitors that have been developed as anticancer drugs to impose cytostatic and/or cytotoxic effects on cancer cells, when used in doses ten times lower, almost exclusively inhibit mTORC1 similar to Rapamycin. Second, at these low doses, these inhibitors also inhibit Rapamycin-insensitive target 4E-BP that plays an important role in senescence hypertrophy and morphology. In some sense, mTORins look like more attractive drugs than rapalogs when used in low non-cytostatic doses [9]. Although, at these doses mTOR inhibitors (mTORins) also start inhibiting mTORC2, this inhibition is rather minimal: no cytotoxic effects have been observed. This concentration could be called optimal gerosuppressive concentration. Therefore at these concentrations, mTORins may have no more side effects than Rapamycin, although animal experiments will be needed to prove this point (at this moment, the inhibitors were tested only in the cell culture). More importantly, mTORins are more efficient in preventing positive beta-gal staining and flat cell senescence morphology than rapalogs [9]. What is necessary is to define optimal concentration of all mTORins for clinical use. This super gerosuppressive drugs may become new cornerstone in anti-aging drug development.
